# HfS_2_ thin films deposited at room temperature by an emerging technique, solution atomic layer deposition†

**DOI:** 10.1039/d1dt01232k

**Published:** 2021-08-25

**Authors:** Yuanyuan Cao, Sha Zhu, Julien Bachmann

**Affiliations:** Chemistry of Thin Film Materials (CTFM), Interdisciplinary Center of Nanostructured Films (IZNF), Friedrich Alexander University of Erlangen-Nuremberg Cauerstr. 3 91058 Erlangen Germany julien.bachmann@fau.de; Institute of Chemistry, Saint-Petersburg State University Universitetskii pr. 26 St. Petersburg 198504 Russia

## Abstract

As a member of the two-dimensional metal dichalcogenide family, HfS_2_ has emerged as a promising material for various optoelectronic applications. Atomic layer deposition is widely used in microelectronics manufacturing with unique properties in terms of accurate thickness control and high conformality. In this work, a simple and versatile method based on the atomic layer deposition principles is presented to generate hafnium disulfide from the solution phase ('solution ALD' or sALD). For ease of comparison with the traditional gaseous atomic layer deposition (gALD) method, the same precursors are used, namely tetrakis-(dimethylamido) hafnium(iv) and H_2_S. The deposit is characterized on several different oxide substrates by spectroscopic ellipsometry, scanning electron microscopy, and X-ray photoelectron spectroscopy. In the saturated regime, the growth rate depends on the substrate nature and is between 0.4 and 0.6 Å per sALD cycle. This growth rate determined at room temperature is lower than with the gALD process reported at 100 °C recently. At those low deposition temperatures, the films remain in an amorphous state. This success in sALD expands the range of material classes available by the new method, adding transition metal dichalcogenides to the list containing oxides, cubic sulfides, hydrides, and organics so far. It promises to overcome the precursor constraints associated with the traditional gALD method, in particular the volatility requirement.

## Abstract 2

The two-dimensional material and semiconducting dichalcogenide hafnium disulfide is deposited at room temperature by atomic layer deposition from molecular precursors dissolved in hexane.

## Graphical abstract

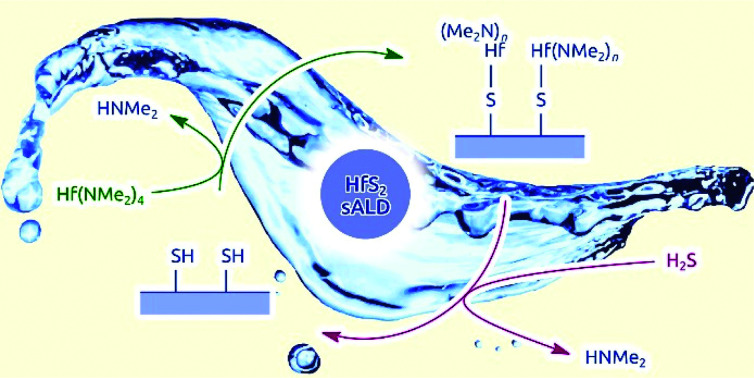

## Introduction

HfS_2_, as a member of the transition metal dichalcogenides, has recently emerged as a promising material for electronics and energy conversion applications in the semiconductor community due to its sizeable bandgap and other favorable physical properties.^1–9^ In comparison to its bulk counterpart, thin film HfS_2_ has shown further intriguing properties.^1,10^ The methods reported so far for the synthesis of thin film HfS_2_ have been mechanical exfoliation and chemical vapor deposition.^11–14^ Mechanical exfoliation is not applicable to the systematic variation of film thickness and study of the physical properties as they depend on it. Furthermore, its use in practical applications is debatable. Chemical vapor deposition yields continuous films over sizeable areas, which can be of high quality after treatment at elevated temperature. The film thickness homogeneity, however, becomes limited for large substrates. Accordingly, significant effort has been dedicated to developing atomic layer deposition methods for the generation of HfS_2_ thin films over large areas.^15–17^ Atomic layer deposition (ALD) relies on self-limiting surface chemical reactions of two distinct precursors introduced in the vapor phase to react with the solid surface in a sequential manner, avoiding direct contact between both precursors. Repeating the alternating pulses of the two precursors allows one to deposit films with high conformality even in deep pores and with accurate thickness control.^18^ In this traditional, gas-based ALD variant (gALD), the precursors need to fulfill several characteristics simultaneously: volatility, reactivity, and thermal stability to avoid thermal decomposition.^19,20^ These requirements constrain the list of ALD-grown materials. Moreover, vacuum conditions and high processing temperatures limit the range of thin film materials and substrates for which it is adequate.

Recently, the availability of deposition methods relying on self-limiting surface chemical principles^21–27^ inspired the development of solution atomic layer deposition (sALD) as a general equivalent of gALD in solution processing.^28–31^ The simplicity of its experimental realization represents one additional advantage of the novel method. Here, the surface chemical reactions of the precursors need to provide sufficient driving force for the formation of the desired materials, as in gALD, but further constraints are eliminated, in particular the volatility. Furthermore, a wider variety of reactivity types can be exploited which are not accessible from the gas phase, such as those using ionic reagents or large organic molecules. We have also observed that many sALD reactions can be run even at room temperature, perhaps aided by the presence of the solvent. After demonstrating the sALD generation of oxides^32^ and a cubic sulfide,^33^ we have started expanding sALD beyond the confines of gALD materials families with a hydride^34^ and a polymeric solid.^35^ This study demonstrates the applicability of sALD to yet another interesting family of materials, namely transition metal dichalcogenides (TMDCs). We focus on HfS_2_ from the precursors Hf(NMe_2_)_4_ and H_2_S used recently in gALD. We establish the successful formation of continuous closed layers of HfS_2_ by sALD at room temperature, with a growth rate of approximately 0.5 angstrom per cycle. Proving that a given gALD reaction can also implemented in sALD with similar characteristics emphasizes the indispensability of considering sALD as an integral part of the ALD family of techniques.

## Experimental section

### Chemicals

Hf(NMe_2_)_4_, H_2_S solution (0.8 M in tetrahydrofuran), *n*-hexane, and CaCl_2_ were ordered from Abcr, Sigma, and VWR. All the chemicals were used as received if there is no further clarification. Peristaltic pumps are of model REGLO ICC from ISMATEC. Tube connections were supplied by Waston-Marlow (Viton solvent resistant tubing) with 1.52 mm inner diameter. Silicon (100) wafers with a 200 nm thermal SiO_2_ layer were purchased from Silicon Material Inc. The purchased *n*-hexane was treated with anhydrous CaCl_2_ and rotivaped, then stored in the moisture-free N_2_ glovebox with 3/4 Å molecular sieves for the further use.

### Handling of the Hf(NMe_2_)_4_

Hf(NMe_2_)_4_ is highly sensitive to moisture and oxygen. To avoid any contact with substances that will cause it to decompose, the precursor solutions and solvent were prepared under inert atmosphere and then transferred to a N_2_ Schlenk line for the further processing.

### sALD technical details

The information in the main manuscript text is complemented by the following details. The Teflon tubes for precursor delivery are connected to the sALD chamber with threaded screw sets and ferrules, and at their other extremity the tubes are connected to the flasks with cannulae. The elastomer tubes tested in the peristaltic pumps include Viton (black) and Matson Marlow's solvent-resistant product 984.0152.000 (yellow). The list of solvents considered is presented in the ESI (Table S1†), as are the solids obtained by direct reaction of precursors with each other (Fig. S1 and S2†).

### Material characterizations

The film thickness was measured by the SENPro spectroscopic ellipsometer from SENTECH. The measurements were carried out at an angle of 70°, and on a spectral range of 370–1050 nm. The film thickness was fitted with a model consisting of air/HfS_2_/SiO_2_/Si stacks. The crystal structure of the deposit was characterized by X-ray diffraction (XRD) using a Bruker (Germany) D8 advance diffractometer equipped with a Cu Kα radiation source and a LynxEye XE-T detector. Energy dispersive X-ray spectroscopy (EDX) and the corresponding scanning electron microscopy images were acquired using a JEOL (Japan) JSM 6400 PC system equipped with a LaB_6_ cathode and SDD X-ray detector. All other SEM images were acquired using a Zeiss (Germany) Gemini 500. X-ray photoelectron spectroscopy (XPS) spectra were recorded with monochromatized Al K_α_ radiation (PHI Quantera II, Japan), all the spectra were calibrated with the C 1s binding energy 284.6 eV. A quartz crystal microbalance (QCM, purchased from Novaetech Srl) was used for the determination of the *in situ* growth of HfS_2_.

## Results

### sALD setup

**Fig. 1:**
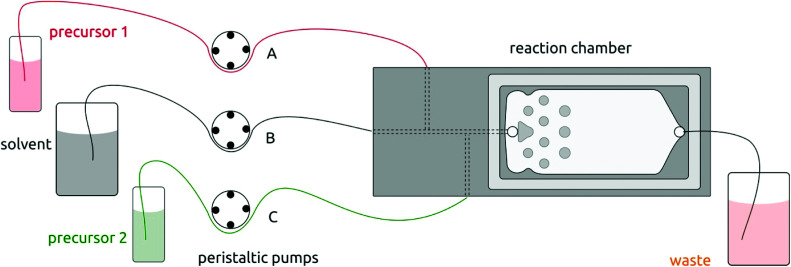
Schematic illustration of the solution atomic layer deposition (sALD) setup. Three peristaltic pump channels drive the precursors and solvent to the reaction chamber in alternating fashion.

The setup for solution atomic layer deposition is sketched in Fig. 1. The sample substrates are loaded in the microfluidic reaction chamber, which is made of stainless steel. A glass slide (2.5 cm × 7.5 cm) is used to secure the chamber with an O-ring sealing. Teflon tubes connect the chamber with the precursor and solvent flasks (which consist of classic Schlenk glassware for air-sensitive chemistry). Peristaltic pumps deliver precursors into the chamber *via* both lateral channels in alternating manner. These pulses of precursors 1 and 2 are separated by purges during which pure solvent is injected *via* the central channel only. The pure solvent pump is always on in order to deliver the equivalent of a ‘carrier gas’ in gALD.

### Preliminary tests

Hf(NMe_2_)_4_ and H_2_S solution (0.8 M in THF) are used as hafnium(iv) and sulfide sources for the HfS_2_ solution atomic layer deposition (sALD). The solvent choice is dictated by the following requirements: stability of both precursors to it, reactivity of precursors to each other in it, and stability of the tubing to it. Hexane fulfills those requirements and is used to dissolve (or dilute) both precursors to 1 or 2 mM based on the stoichiometry of the desired solid. When both precursor solutions are mixed directly, a yellow precipitate is formed, which maintains its color for weeks under nitrogen but loses its color upon exposure to air. Accordingly, precursors and products must be handled under strictly inert atmosphere.

### sALD study

The main parameters that can be tuned in sALD are precursor concentrations, solution flow rates, as well as precursor pulses and purge durations. Together, these parameters must provide sufficient precursor delivery to the surface, avoid direct precursor contact, and minimize solvent consumption, so that an efficient trade-off must be found between those specific constraints.

As substrates for this study, we will consider various oxides in order to address potential nucleation difficulties: Si wafers with 200 nm thermal SiO_2_ and optionally coated with either SnO_2_, Al_2_O_3_, or ZnO (by ALD). Anhydrous *n*-hexane is used as the purging solvent. In a first control experiment, no solid is deposited when a hexane solution of Hf(NMe_2_)_4_ flows over the substrate in the absence of complementary reagent for the equivalent of 30 sALD cycles. Fig. S3 in the ESI† exhibits the formation of a layer on the order of 0.1 nm thickness, corresponding to the one adsorbed monolayer of Hf complex that must be expected. This demonstrates that Hf(NMe_2_)_4_ does not decompose thermally or in the presence of adventitious water.

**Fig. 2:**
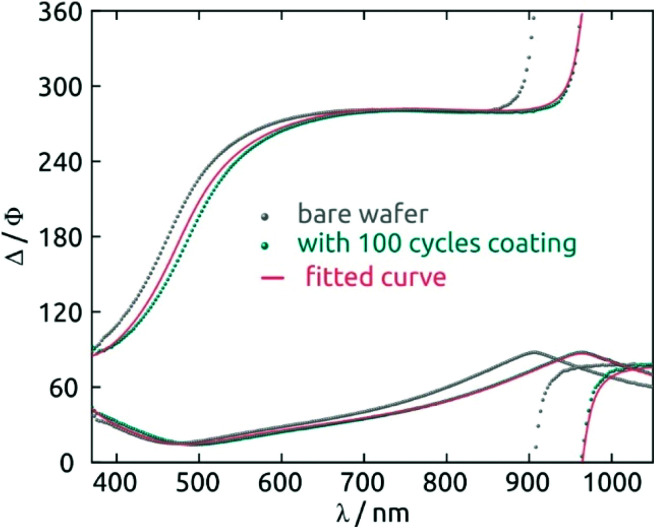
Hafnium sulfide film thickness characterization: spectroscopic ellipsometry data obtained from a bare silicon wafer (in gray), 100 cycles HfS_2_ coated wafer (in dark cyan), and curves fitted with an air/HfS_2_/SiO_2_/Si stacks model (in pink).

For deposition tests, 1 mM Hf(NMe_2_)_4_ and 2 mM H_2_S *n*-hexane solutions prepared under inert atmosphere are injected into the chamber in alternating manner, after a preliminary flush with pure solvent. One standard sALD cycle is defined as follows: (i) Hf(NMe_2_)_4_ solution is pumped into the chamber for 10 s. (ii) The chamber is purged with *n*-hexane for 60 s. (iii) The H_2_S solution is pumped for 10 s. (iv) The chamber is purged again for 60 s. After the desired number of cycles, *n*-hexane is used for a final purge for 3 minutes. After a 100-cycle test, the thickness of the layer determined by spectroscopic ellipsometry is 5.8 nm (Fig. 2). Samples look homogeneous even after 410 cycles (Fig. S4a†).

**Fig. 3:**
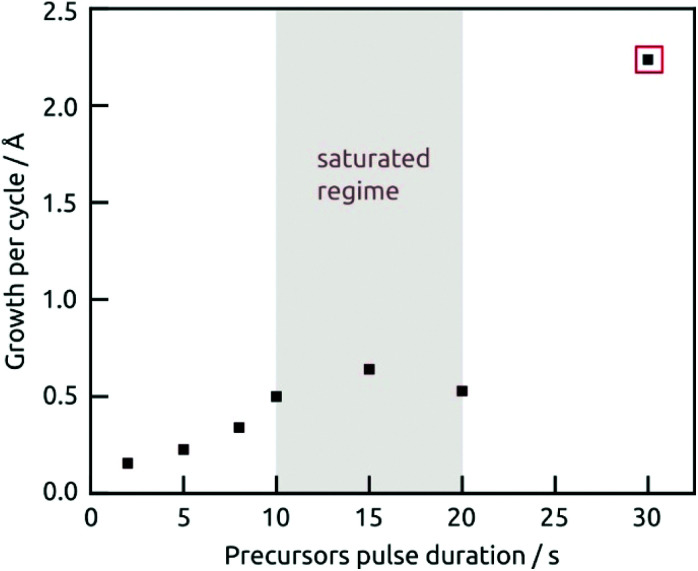
Growth rate as a function of the precursors pulse duration, the concentrations for Hf(NMe_2_)_4_ and H_2_S are 1 mM and 2 mM, respectively, and the solvent purging is 60 s for each half cycle. Note that the thickness for the marked data point was measured after removing the dusty precipitate with sonication in hexane.

**Fig. 4:**
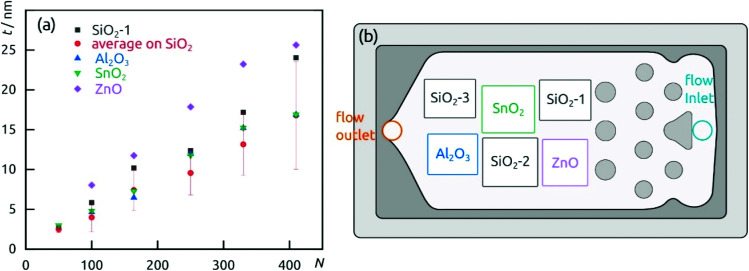
(a) Film thickness dependence on the number of cycles performed on different substrates. (b) Wafers distribution in the reaction chamber.

The demonstration of self-limiting surface chemistry which defines ALD growth is provided by a series of tests in which the precursor pulse duration is varied (while the purge duration is maintained, Fig. 3). The saturating behavior is found for pulses of 10 to 20 s, proving ALD growth. For extreme pulse durations, the purge no longer suffices to prevent uncontrolled deposition (of the chemical vapor deposition or chemical bath deposition type, last datapoint of Fig. 3 and Fig. S4b†). The linear growth behavior is demonstrated on the four types of substrates tested in Fig. 4. In this experiment, care must be taken to reproduce the same placement of substrates in each individual run (Fig. 4b) in order to account for deviations in growth rate due to placement and substrate chemistry. Although all substrates exhibit linear growth, the growth rate varies significantly, with samples situated close to the inlet experiencing more deposition. This observation indicates that the flow dynamics of the microfluidic chamber design can be improved further. In addition to this, ZnO seems to give rise to a slightly faster growth, perhaps associated with its higher surface roughness.

### Characterization of the deposit

**Fig. 5:**
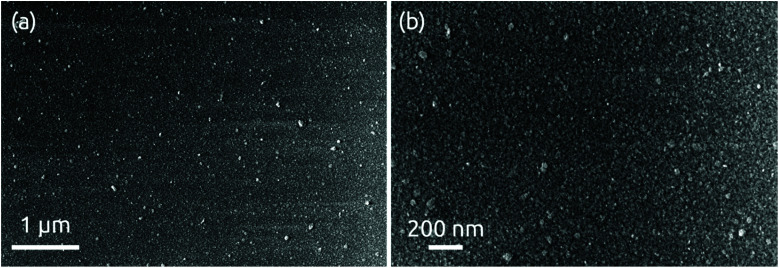
SEM images of 24 nm of HfS_2_ deposition grown on a bare Si/SiO_2_ wafer at (a) low and (b) high magnification levels.

The morphology of a 24 nm HfS_2_ film deposited on a bare wafer is continuous with some roughness observable in scanning electron micrographs, Fig. 5. X-ray diffraction measurements performed in grazing incidence do not yield any hint of a crystalline structure (Fig. S6†). We conclude that the deposit is amorphous, as expected given the very low processing temperature.

**Fig. 6:**
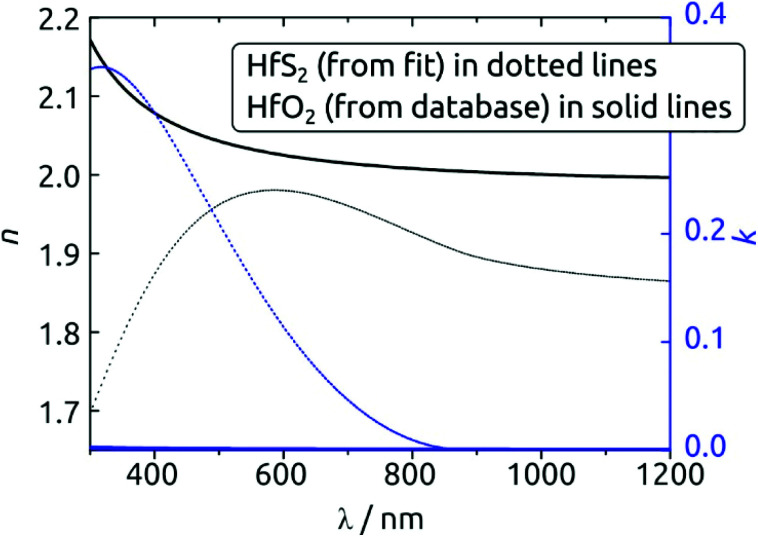
Refractive index and extinction coefficient spectra obtained for the HfS_2_ deposition (dotted lines), as compared with the database reference values for HfO_2_ (solid lines). The clearly distinct curves furnish a strong argument for the chemical identity of the deposit.

A strong indication for the identity of the deposit as HfS_2_ is provided by the spectroscopic ellipsometry data. Fig. 6 exhibits the *n*(*λ*) and *k*(*λ*) spectra obtained from the fit to the experimental data. As a comparison, we also provide the database spectra of HfO_2_. The material deposited is clearly not HfO_2_. Not only do the refractive indices diverge, especially in the UV, but the solid deposited with our method absorbs strongly over a large fraction of the visible range, whereas HfO_2_ is of course perfectly transparent. The bandgap obtained from the Tauc-Lorentz optical model is 1.4 eV, a value smaller than that reported for crystalline HfS_2_ (1.8 eV)^36^ but possible given the amorphous nature of our material and the range of values computed for different configurations of HfS_2_.^37^

**Fig. 7:**
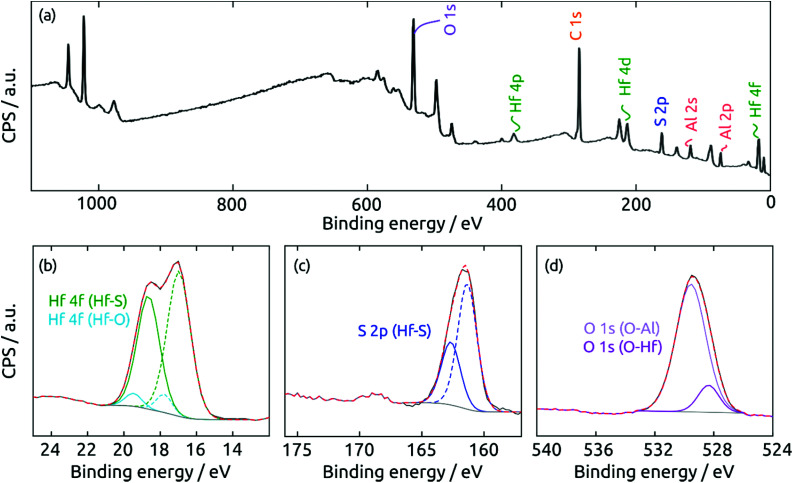
XPS spectra of a HfS_2_ film on a Al_2_O_3_ substrate. (a) Survey spectrum, (b) Hf 4f, (c) S 2p, and (d) O 1s regions.

The surface composition of the coating exposed to air is determined by X-ray photoelectron spectroscopy (XPS). The survey spectrum (Fig. 7a) of a HfS_2_/Al_2_O_3_/SiO_2_/Si sample exhibits the desired elements Hf and S, together with Al and O from the substrate. Not surprisingly, the surface is partly oxidized in air. The high-resolution Hf 4f region in Fig. 7b can be deconvoluted into two doublets (Hf 4f_7/2_/4f_5/2_) centered at 17.0 and 18.6 eV and at 17.8 and 19.3 eV, which can be ascribed to Hf bonded to sulfide and to oxide, respectively. The S 2p region in Fig. 7c can be fitted to two components at 161.3 and 162.7 eV, which are associated with the 2p_3/2_/2p_1/2_ doublets of sulfide in HfS_2_. Finally, the O 1s region in Fig. 7d confirms the presence of HfO_2_ (O 1s at 528.4 eV) and Al_2_O_3_ (529.6 eV). To sum up, the XPS measurements prove the success of HfS_2_ growth by sALD, and remind us of the highly oxyphilic nature of the element Hf.^16,38–40^

## Discussion

A comparison of the HfS_2_ sALD results with our gALD process published recently provides interesting insight.

The growth rate in this sALD is 0.4 Å–0.6 Å per cycle at room temperature. Compared to the gALD process (1.2 Å per cycle at 100 °C in gALD),^16^ this value is significantly smaller. This result points to an influence of the solvent on the surface reactions. A molecular-level explanation of this effect is suggested by the somewhat unusual shape of the growth rate dependence on temperature in gALD (shown in Fig. 3 of that paper).^16^ Indeed, a constant growth rate of 1.2 Å per cycle from 65 °C to 100 °C is followed by another plateau from 130 °C to 180 °C at the lower value 0.6 Å per cycle in gALD. This behavior contrasts with the most common curve, which typically combines a plateau up to the decomposition temperature of a precursor, after which the growth rate increases. The facts that in gALD (a) two seemingly self- saturating regimes exist, and (b) the high-temperature regime exhibits half of the growth rate obtained at lower temperature may be related to the formation of a Hf(NMe_2_)_4_ dimer, the stability of which is observed in DFT computations (ESI of the publication).^16^ This dimer could form on the surface during gALD growth at low temperature and be responsible for the elevated growth rate. It would, however, be disrupted by temperatures in excess of 100 °C. Interestingly, the formation of this dimer seems to also be prevented at low temperature by the presence of solvent.

On a more general level, our results show that obtaining crystalline TMDCs at low temperature remains an elusive target. Furthermore, the extreme air sensitivity of HfS_2_ may prevent it from being the best suited model system in which to study the details of nucleation and growth. However, the fact that one ALD reaction can be performed both from the gas phase and the liquid phase demonstrates the continuity that exists between sALD and gALD, and it opens new avenues of research towards low-temperature deposition of crystalline 2D materials. Indeed, the fine-tuning of interfacial energies is crucial for the control of the film morphology, and it can be adjusted best from the liquid phase. To this goal, the choice of solvents, the variation of ligands in the metal–organic precursors, and the use of additives such as surfactants represent tools that are available to the experimentalist from the gas phase only.

## Conclusions

In this study, we developed an sALD method for the generation of HfS_2_ thin films from Hf(NMe_2_)_4_ and H_2_S as precursors. The chemical reaction is the same used in gas-ALD already and yields similar results. The data presented in this paper establish the applicability of sALD to deposit transition metal dichalcogenides (which in crystalline form are 2D materials). It also provides one additional example of sALD as one integral part of the ALD family of techniques. The advantages of sALD include the simplicity and affordability of the setup, the low precursor consumption, as well as recyclability of solvents. Importantly, the volatility requirement on gALD precursors is released in sALD, which expands the palette of applicable precursors, and eventually broadens the type of materials achievable *via* atomic layer processing.

Coming back to the specific reaction presented here, XPS demonstrates the success formation of HfS_2_, whereas the films are amorphous due to the low processing temperature. The comparison of sALD characteristics with the gALD process demonstrates that the growth is significantly slower in sALD (0.4 Å to 0.6 Å per cycle at room temperature *vs.* 1.2 Å per cycle at 100 °C in gALD). This result points to an influence of the solvent on the surface reactions. In our opinion, this example provides a hint that the solvent in sALD must be considered as more than just a bothersome potential source of impurities. Rather, it should be seen as an additional tool that the experimentalist can exploit in sALD to influence surface chemistry and select reactive pathways at will.

## Author contributions

YC performed most of the investigation and wrote the original draft. SZ helped with the preliminary tests and prepared the sample for XPS measurements. JB contributed conceptualization and manuscript review and editing. YC and JB performed data curation.

## Conflicts of interest

The authors declare no competing financial interest.

## Supplementary Material

DT-050-D1DT01232K-s001
