# Supplementary material for: HfS2 thin films deposited at room temperature by an emerging technique, solution atomic layer deposition
Source: Dalton Trans. 2021 Aug 25;50(37):13066–72. doi: 10.1039/d1dt01232k (PMC8477444; doi:10.1039/d1dt01232k)
Supplement: DT-050-D1DT01232K-s001 [file DT-050-D1DT01232K-s001.pdf]

Electronic Supplementary Material (ESI) for Dalton Transactions.
This journal is © The Royal Society of Chemistry 2021

Supplementary Information

for

HfS_2 Thin Films Deposited at Room Temperature by an Emerging Technique, Solution Atomic Layer Deposition

Yuanyuan Cao,[1] Sha Zhu,[1] Julien Bachmann[1,2]

1. Chemistry of Thin Film Materials (CTFM), Interdisciplinary Center of Nanostructured Films (IZNF), Friedrich Alexander University of Erlangen-Nuremberg, Cauerstr. 3, 91058 Erlangen, Germany
2. Institute of Chemistry, Saint-Petersburg State University, Universitetskii pr. 26, St. Petersburg 198504, Russia

E-mail: julien.bachmann@fau.de

Table S1. Tube compatibility and precursors reactivity in different solvents.

Solvent	tubes compatibility in the solvent		$Hf(NMe_2)_4$	
	yellow	black (Viton)	solubility	reactivity with H_2S
THF[a]	severe damage	severe damage	yes	yes
DE[b]	fair	fair	yes	no
toluene	severe damage	good	yes	no
DE+toluene	severe damage	fair	yes	yes
hexane	good	good	yes	yes
hexane+toluene	good	good	yes	yes
DCM[c]	severe damage	good	yes	yes
DCM+toluene	severe damage	good	yes	yes

[a] tetrahydrofuran, [b] diethyl ether, and [c] dichloromethane

Since the sALD technique is not only limited by the driving force of the two precursors to for the desired materials, but also the compatibility of the tubes involved in the peristaltic pumps with the purging solvent. Thus we also tested the tube resistance in different solvents, as it is shown in the **Table S1**, $Hf(NMe_2)_4$ is soluble in all the solvents and solvent mixtures we tested. However, the two precursors cannot react with each other either in pure diethyl ether or in pure toluene, which means those two solvents cannot be used as purging solvent. Interestingly, the mixture of diethyl ether and toluene can be used as a flushing solvent as the two precursors do react with each other in the mixture. THF damages the two commercial tubes we have for the peristaltic pumps. All others listed in the table can be used as the flushing solvent for our HfS_2 sALD because they are all inert to at least one of the tubes and in which the precursors are reactive. In those solvents we can observe an immediate color change from colorless to yellow and the formation of the precipitation after mixing the two precursors (**Figure S1**).

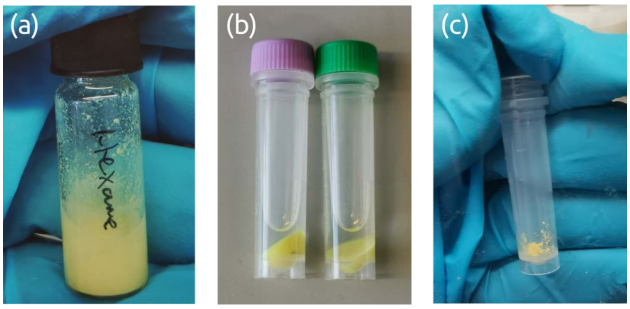

Figure S1. Photographs taken of the precipitate obtained up mixing $Hf(NMe_2)_4$ and H_2S. (a) Immediately after the mixing. (b) After centrifugation. (c) After washing with n-hexane, centrifugation 3 times and drying under vacuum overnight.

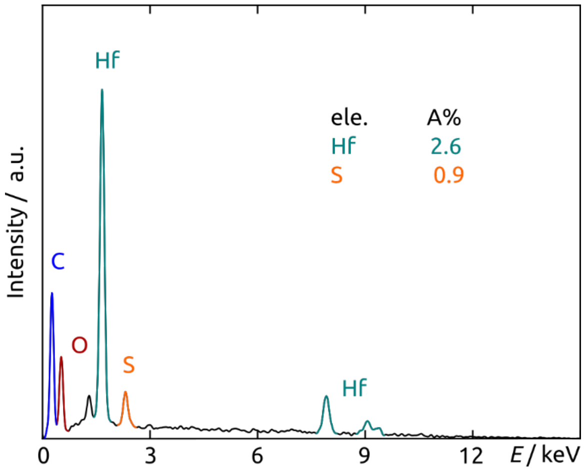

Figure S2. EDX spectrum on the precipitate obtained from the direct reaction of $Hf(NMe_2)_4$ and H_2S in n-hexane.

The precipitation obtained from the preliminary direct mixing of the two precursors can stay yellowish for weeks in the nitrogen atmosphere. After removing the solvent and washing the precipitate several times, we obtained the yellowish powder (**Figure S1**). EDX measurement was performed on the powder (**Figure S2**), however, we observed substoichiometric Hf to S ratio highly due to the instability of HfS_2 in ambient which has been widely reported.[s1-s3] The color of the precipitate gradually turned to white after exposure to air. Thus, it is of high importance to avoid leakage to protect the Hf precursor as well as the product.

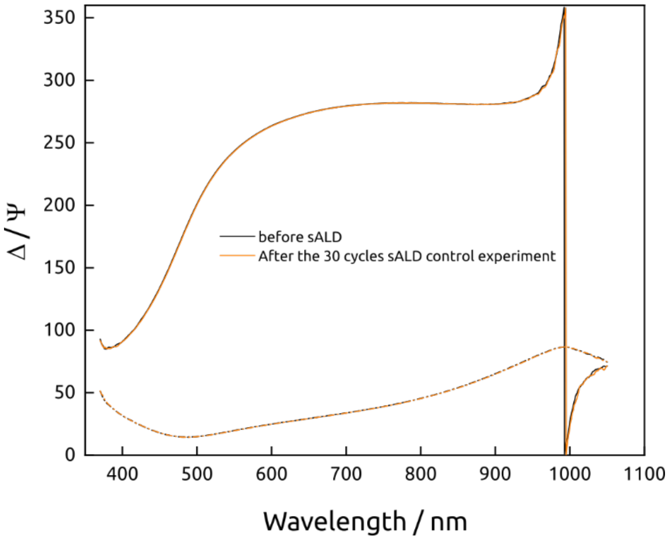

Figure S3. Spectroscopic ellipsometry curves before and after 30 cycles control experiment run on the SnO_2 coated wafer without H_2S precursor. The sequence is 10 s 2.5 mM $Hf(NMe_2)_4$ pulse / 60 s hexane purge / 10 s hexane pulse / 60 s hexane purge.

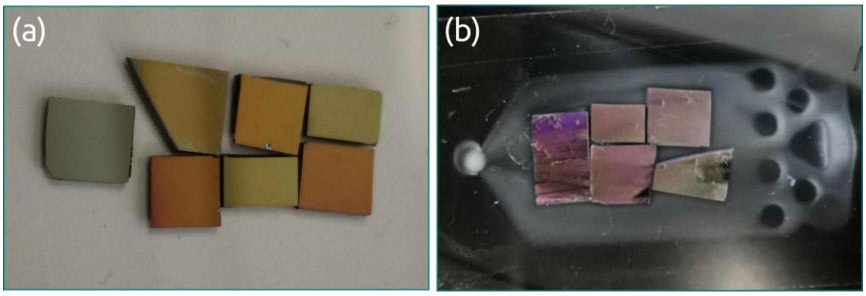

Figure S4. A comparison of photographs of the ALD (a) and CBD (b) type growth. (a) Photo of the substrates after 410 cycles sALD. Recipe: $Hf(NMe_2)_4$ and H_2S were 1 mM and 2 mM, respectively. 10 s pulse, 60 s purge. (b) Photo of the wafers after 50 cycles sALD with 30 s pulse / 60 s purge.

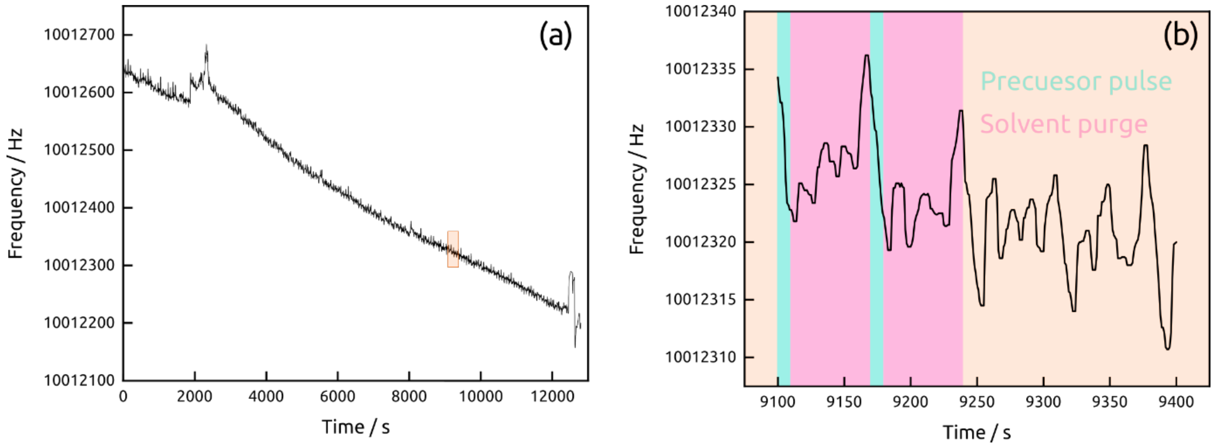

Figure S5. Quartz crystal microbalance data recorded on the sALD sequence of 10 s precursors pulse / 60 s solvent purge for 100 cycles. The crystal has an eigenfrequency of ~10 MHz. Step for data recording: 1 s per data point.

From the QCM data, we observed a clear linear frequency decrease trend along the 100 cycles sequence, and the frequency change is periodically consistent with the cycle length (140 s). A clear plateau behavior of the QCM data as normally expected in gALD is overserved here.

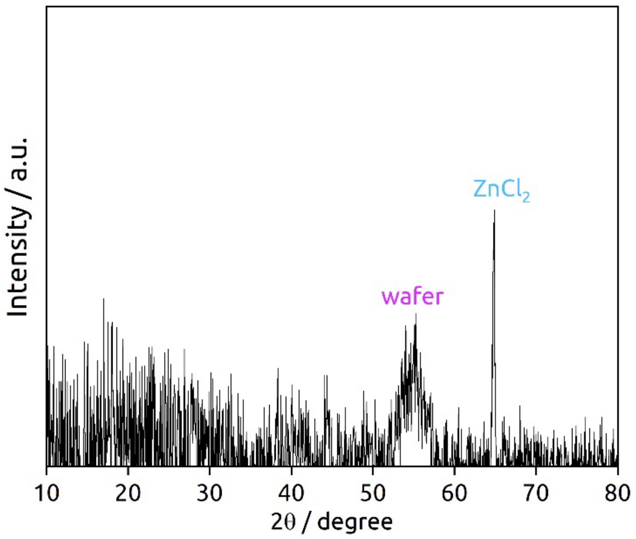

Figure S6. Grazing-incidence X-ray diffraction (GI-XRD) pattern of 150 cycles HfS_2 sALD with a ZnS protective layer. ALD recipe for HfS_2 deposit: 10 s $Hf(NMe_2)_4$ (1 mM) pulse / 60 s purge / 10 s H_2S (2 mM) pulse / 60 s purge, 150 cycles. And the recipe for the ZnS capping layer: 10 s $ZnCl_2$ (2 mM) pulse / 20 s purge / 10 s H_2S (2 mM) pulse / 20 s purge, 100 cycles.

GI-XRD was used to check the crystallinity of the HfS_2 deposit. Since HfS_2 is a sensitive material, we performed a deposition of ZnS from $ZnCl_2$ (2 mM in a toluene / hexane mixture) and H_2S (2 mM) solutions as a capping layer. For the capping layer, the choice is not limited to ALD grown materials, considering the time cost and solvent consumption, we performed the ZnS capping in a chemical bath deposition fashion. The XRD result shows only peaks from the substrate,[s4] and a peak from the Zn source for the ZnS layer. Essentially, the HfS_2 deposit is in amorphous phase.

Reference

s1. Q. Li, L. Shi, R. Wu, C. Lin, X. Bai, Y. Ouyang, B. A. Baraiya, P. K. Jha and J. Wang, *Phys. Chem. Chem. Phys.*, 2019, **21**, 17010-17017.
s2. G. Mirabelli, C. McGeough, M. Schmidt, E. K. McCarthy, S. Monaghan, I. M. Povey, M. McCarthy, F. Gity, R. Nagle, G. Hughes, A. Cafolla, P. K. Hurley and R. Duffy, *J. Appl. Phys.*, 2016, **120**, 125102.
s3. S. H. Chae, Y. Jin, T. S. Kim, D. S. Chung, H. Na, H. Nam, H. Kim, D. J. Perello, H. Y. Jeong, T. H. Ly and Y. H. Lee, *ACS Nano*, 2016, **10**, 1309-1316.
s4. Y. Cao, Y. Wu, C. Badie, S. Cadot, C. Camp, E. A. Quadrelli and J. Bachmann, *ACS Omega*, 2019, **4**, 8816-8823.
